# Three-dimensional measurement of the depth of invasion in oral squamous cell carcinoma samples using Lugol's iodine-enhanced micro-computed tomography: an original study

**DOI:** 10.1590/1678-7757-2024-0304

**Published:** 2024-01-10

**Authors:** Jiaxin Yu, Zhouyu Gu, Lichan Wang, Qian Zhang, Yumei Pu, Qingang Hu, Chengwan Xia, Yuxin Wang

**Affiliations:** 1 Nanjing University Research Institute of Stomatology Affiliated Hospital of Medical School Nanjing China Nanjing University, Research Institute of Stomatology, Affiliated Hospital of Medical School, Nanjing Stomatological Hospital, Department of Oral and Maxillofacial Trauma Orthognathic Plastic Surgery, Nanjing, China.; 2 Nanjing University Research Institute of Stomatology Affiliated Hospital of Medical School Nanjing China Nanjing University, Research Institute of Stomatology, Affiliated Hospital of Medical School, Nanjing Stomatological Hospital, Department of Oral and Maxillofacial Head and Neck Oncology Surgery, Nanjing, China.

**Keywords:** Micro-computed Tomography, Lugol's Iodine, Oral squamous cell carcinoma, Depth of invasion

## Abstract

**Objectives:**

Depth of invasion (DOI) in oral squamous cell carcinoma (OSCC) guides treatment and prognosis but lacks three-dimensional (3D) insight. Thus, this study aimed to investigate the feasibility and accuracy of Lugol's iodine-enhanced micro-computed tomography (CT) for the 3D measurement of DOI in OSCC samples.

**Methodology:**

In total, 50 *in vitro* OSCC samples from Nanjing Stomatological Hospital (July 2022 to January 2024) were subjected to micro-CT imaging with a slice thickness of 50 μm following 3% Lugol iodine staining for 12 h, followed by pathological examination and staining. The panoramic diagnostic scanner digitally measured pathological DOI. The micro-CT DOI was measured by evaluating the voxel value of the boundary of the tumor lesion and comparing it with the pathological examination results. Experienced physicians analyzed both measurements, and statistical analyses were performed to determine their correlation.

**Results:**

Lugol iodine-enhanced micro-CT imaging distinguishes various tissue structures, such as tumor tissue, epithelial tissue, muscle tissue, blood vessel structure, and other major tissue structures in 3D space. This imaging technique found and localized micro-tumor lesions (1.82×1.5×1 mm^3^) when in conjunction with pathological sections. Statistical analysis indicated a strong correlation between pathological DOI and micro-CT DOI (*P*<.001; r=0.986). During DOI measurement, Lugol iodine-enhanced micro-CT imaging effectively compensated for the loss of 3D space information in the pathological measurements, improving the accuracy of the DOI measurement.

**Conclusions:**

Lugol iodine-enhanced micro-CT improves OSCC DOI 3D measurements, enhances pathological staging accuracy, and aids treatment decisions and prognosis.

## Introduction

Oral squamous cell carcinoma (OSCC) is the most prevalent malignant tumor of the head and neck, significantly impacting patients’ quality of life. Recent statistics estimate that, by 2024, about 58,450 new cases and 12,230 deaths will be attributed to oral cancer globally, with OSCC representing from 80 to 90% of these cases.^1^ Depth of invasion (DOI) consists of the distance from the basement membrane to the deepest point of tumor infiltration.^2^ Patients showing a high DOI are associated with an increased rate of regional lymph node metastasis and poorer overall survival following surgical intervention.^3^ The eighth edition of the American Joint Committee on Cancer (AJCC) guidelines has incorporated DOI into its pathological T-staging assessment for OSCC.^4^ Therefore, precise measurement of DOI is crucial for effective OSCC management.

Currently, pathological examination remains the gold standard to assess DOI. However, this method typically involves microscopic evaluations in which pathologists rely on their expertise to obtain extensive sections from the deepest areas of tumor infiltration. This slicing technique often loses three-dimensional (3D) information and can underestimate DOI values. While serial sectioning by whole tumor samples is considered optimal to measure DOI,^5^ it poses practical challenges due to high costs, time demands, and potential sample destruction in clinical settings. Commonly employed imaging modalities such as computed tomography (CT) and magnetic resonance imaging (MRI)^6^ serve as alternative methods to estimate DOI. However, spatial resolution and density limitations may compromise their accuracy.

Micro-CT imaging offers high-spatial-resolution three-dimensional information regarding biological tissues.^7^ Additionally, moderate Lugol iodine staining addresses issues related to low-density resolution.^8^ Previous research indicates that micro-CT enhanced with Lugol iodine can effectively differentiate OSCC tissues from normal counterparts.^9^ Nevertheless, its efficacy in accurately measuring DOI remains unexplored.

This study aims to evaluate both the feasibility and precision of using micro-CT to measure DOI in OSCC specimens. Furthermore, we seek to show that Lugol's iodine-enhanced micro-CT imaging more accurately assess DOI values than traditional pathological evaluations (which often causes underestimates). Enhanced measurement techniques could facilitate improved TNM staging accuracy and offer better treatment selection^10^ and prognostic assessments^11^ for patients diagnosed with OSCC.

## Methodology

### *In vitro* OSCC tumor sample collection

**Figure 1:**
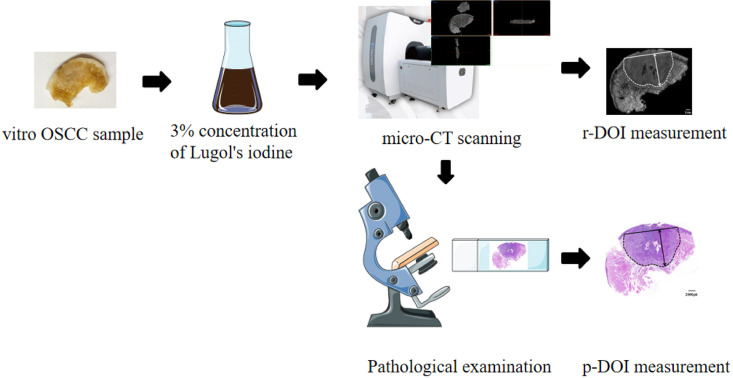
Lugol's iodine-enhanced micro-CT three-dimensional measurement DOI research flow chart.

Patients with OSCC who were admitted to the Department of Maxillofacial Surgery, Stomatology Hospital Affiliated to Nanjing University School of Medicine from July 2022 to January 2024 were selected. This research was conducted in accordance with the Declaration of Helsinki. All 50 patients agreed to participate and signed an informed consent form. Samples of surgically incised lesions were obtained from the pathology department to ensure pathological diagnosis and the remaining marginal lesions between the tumor and normal tissues were subsequently collected for sampling. The samples were required to preserve intact longitudinal sections of the tumor and normal mucosal margins on both sides of the tumor. The collected samples were fixed in a neutral formalin solution. This study was approved by the relevant Medical Ethics Committee (approval number: NJSH-2021NL-030). The clinical trial process is shown in Figure 1.

The *in vitro* OSCC samples were fully soaked in freshly prepared 3% Lugol's iodine for 12 hours. Subsequently, the samples were scanned using micro-CT. After scanning, the samples were digitally scanned following the routine pathological section production process and the r-DOI and p-DOI were respectively measured.

### Patient selection

The following inclusion criteria were chosen: 1) primary OSCC, 2) surgical treatment or surgery + postoperative adjuvant therapy, and 3) preoperative biopsy and postoperative histological confirmation of pathological diagnosis. The following exclusion criteria were chosen: 1) chemotherapy and radiotherapy before surgery; 2) a second operation with metastasis and recurrence; 3) a pathology report that was unable to be evaluated; and 4) a small number of surgical or biopsy specimens potentially affecting the routine pathology diagnosis if performed in the experiment.

### Lugol's iodine-enhanced micro-CT imaging

Lugol (Solarbio, Beijing Solarbio Science & Technology Co., Ltd., Beijing, China) was used as an iodine standard solution with an atomic concentration of 15%. A 10 ml Lugol iodine stock solution was diluted with 40 ml of 1X phosphate-buffered saline (Servicebio, Wuhan Servicebio Technology Co., Ltd., Wuhan, China) to prepare a 3% Lugol iodine solution. Subsequently, the collected OSCC samples were completely immersed in freshly prepared 3% Lugol's iodine for sample staining. Micro-CT imaging (Hiscan XM, Suzhou Haisfield Information Technology Co., Ltd., Suzhou, China) was performed 12 h later. The following scan parameters were chosen: an 8-W power, a 60-kV voltage, a 133.3-A electric current, a 2×2-binging detector mode, a 50-m slice thickness, and a 75-ms repetition time. A 50×40-mm field of view was used. All micro-CT image data were reconstructed and analyzed on SeProcessPro, version 1 (Suzhou Haysfield Information Technology Co., Ltd., Suzhou, China), provided by the manufacturer. Micro-CT — rather than showing the image in a CT value — has no standardization such as a medical CT. Thus, we use the voxel value instead of CT value to describe the resolution of micro-CT.

### Digital pathology section image acquisition

After Lugol's iodine-enhanced micro-CT, all samples were embedded in paraffin using the standard method.^12^ Paraffin-embedded tissue sections (4-μm thickness) were stained and sealed with hematoxylin and eosin by an automatic pathological section staining apparatus. A panoramic diagnostic scanner (Panoramic MIDI, 3D HISTECH Ltd.) was used to scan the slides and obtain digitized pathological section images.

### Measurement of DOI

**Figure 2:**
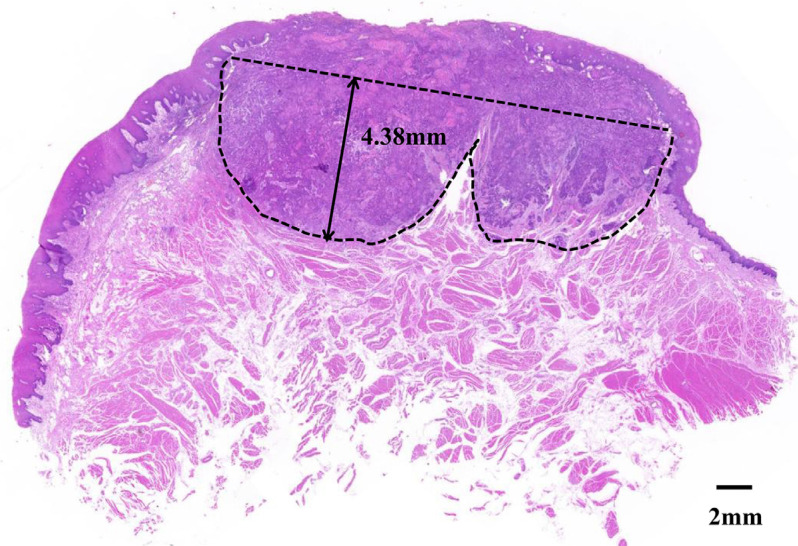
Measuring the depth of invasion from the deepest point of invasion reconstructed basement membrane line.

According to the 8^th^ edition of the AJCC Cancer Staging Manual, DOI consists of the vertical distance from the adjacent normal mucosal basal layer to the forefront of tumor invasion.^13^ Therefore, pathological DOI (p-DOI) was measured based on the hematoxylin and eosin-stained digital pathological section images of the tumor specimens of patients with OSCC. A tangent line was drawn from the basement membrane of the nearest normal mucosal epithelium on both sides of the tumor. The vertical distance from this tangent line to the deepest part of tumor infiltration was defined as the maximum DOI (Figure 2). For exophytic tumors, the exophytic portion of the tumor was excluded from the DOI measurement.^14^ All pathological specimens were evaluated by two experts in head and neck pathology and measured by Caseviewer (V2.4, 3D HISTECH Ltd.). The micro-CT DOI (r-DOI) was measured using the same standard measurement method as above based on Lugol's iodine-enhanced micro-CT images of the tumor specimens of patients with OSCC. The tumor boundary was assessed by measuring the voxel value at the edge of the tumor lesion and comparing it with pathological results. The deepest identifiable infiltration of the tumors in the micro-CT image was subsequently determined. All imaging data were jointly analyzed by two senior head and neck radiologists who were oblivious to patients’ histopathological characteristics and delineated and measured by Mimics Medical (V21.0, Materialize, UK). If the discrepancy between the data obtained by two individuals remained below 1mm, the average of the two was adopted as the final measurement outcome; otherwise, if the discrepancy exceeds 1mm, it will be evaluated by a third expert of the same or higher seniority.

The two-way black arrow solid line represents the depth of invasion (DOI). The dashed line represents the tumor boundary.

### Statistical analysis

SPSS (version 23.0; IBM, Armonk, NY, USA) was used for statistical analyses. Measured data are shown as the mean ± standard deviation. Correlation analysis was performed to determine the closeness and changing trends of the relationship between p-DOI and r-DOI. The correlation coefficient r represents the degree of correlation between p-DOI and r-DOI; the closer r approaches 1, the better the fitting effect of the regression model. All statistical analyses were two-sided, and a *P* value <0.05 was considered statistically significant.

## Results

### Lugol iodine-enhanced micro-CT can clearly distinguish OSCC tumor boundary and fine anatomical structure

**Figure 3:**
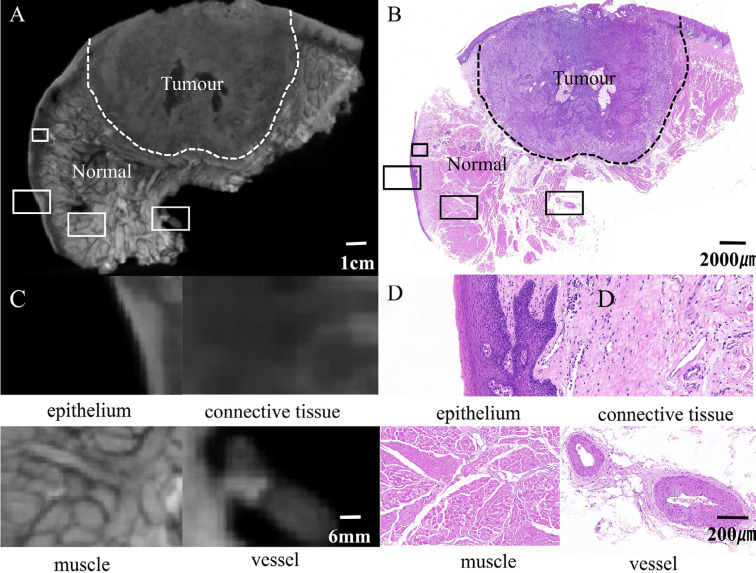
Lugol's iodine-enhanced micro-computed tomography and pathological images of an i*n vitro* sample of oral squamous cell carcinoma. The dashed line represents the tumor boundary. The black boxes correspond to the enlarged parts of the local tissue in the (C) and (D) figures.

This study enrolled 50 patients (36 men and 14 women) to confirm the imaging quality of micro-CT. The average age of patients totaled 62±12 years. They included 24 cases of tongue lesions (20 men and 4 women), 14 buccal lesions (eight men and six women), three mouth floor lesions (all men), six gingival lesions (four men and two women), and three palatal lesions (one man and two women). Results showed that the tumor area on the micro-CT image usually appeared as a uniform and dense solid soft tissue mass image, and the gray value changed slightly between voxels. The histological areas from the same sections showed high consistency (Figure 3A and B), and the measured voxel value in the tumor area totaled 1252.25±11.15 (Table S1). The representative anatomical structures inside normal tissue, including epithelial tissue, muscle tissue, vascular structure, and adipose tissue, can be seen in their characteristic anatomical morphology (Figure 3C-D). Among them, the voxel value of muscle tissue totaled 1361.39±687.64; that of epithelial tissue, 1757.67±898.09; and that of adipose tissue, 678.28±726.99.

(A) Lugol's iodine-enhanced micro-computed tomography (CT) imaging displays the boundary (shown as a white dotted line) between the tumor tissue and normal tissue. (B) Pathological images of the same section. (C-D) Lugol's iodine-enhanced micro-CT images and pathological images of fine anatomical structures of normal tissue, including epithelial, muscle, vascular, and adipose tissue.

### Lugol's iodine-enhanced micro-CT could identify minimal residual lesions

**Figure 4:**
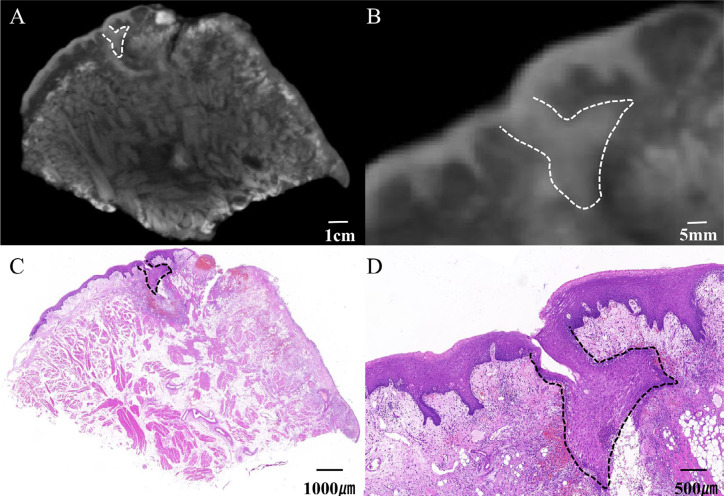
Lugol's iodine-enhanced micro-computed tomography (CT) can identify minimal residual lesions. The dashed line represents the tumor boundary.

This study successfully found a minimal residual lesion (1.82×1.5×1 mm^3^) by Lugol's iodine-enhanced micro-CT. Moreover, comparing the micro-CT and pathological images found the highly consistent tumor boundaries and shape on the micro-CT and pathological images (Figure 4). The pathological sections at the corresponding level showed that the size of the lesion totaled 0.98×1 mm. This shows that micro-CT can accurately locate the tumor margin, which shows the potential value of Lugol's iodine-enhanced micro-CT in tumor resection margin research and provides a strong reference value for clinical whole-tumor lump resection.

(A) The tumor is outlined by a dotted line. (B) Magnification of tumor site on micro-CT. (C) Pathological image of the same section. (D) Magnification of tumor site on pathological section.

### Lugol's iodine-enhanced micro-CT in three dimensions could measure DOI

**Figure 5:**
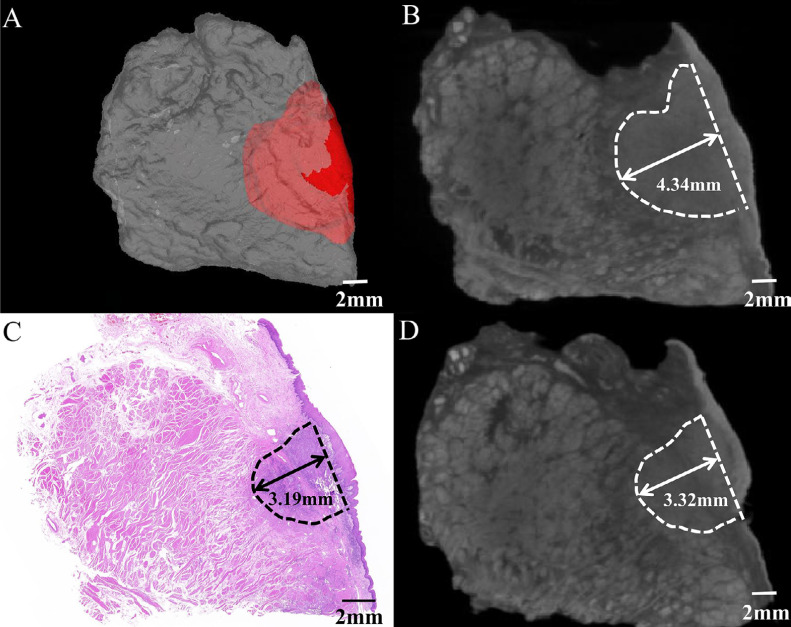
Depth of invasion (DOI) could be measured accurately by Lugol's iodine-enhanced three-dimensional micro-CT imaging. The dashed line represents the tumor boundary. Double arrows with solid black lines indicate DOI.

Pathological measurement configures the gold standard for DOI measurements in OSCC. However, owing to the difficulty of clinical practice, two-dimensional measurements often lead to false results (Figure 5). To explore the feasibility of measuring DOI using Lugol's iodine-enhanced micro-CT in three dimensions, this study included 25 patients with OSCC and the specific clinical information in Table 1. Results showed that the mean value of r-DOI totaled 7.26±2.81 mm; that of the mean value of p-DOI, 6.67±2.70 mm; that of the difference, 0.59±0.39 mm; and that of the mean of 3D r-DOI, 8.34±3.16 mm. Moreover, according to the AJCC8 TNM staging manual, a p-DOI > 5 mm and p-DOI > 10 mm were defined as two key values to estimate OSCC T staging. The DOI results surrounding these two values may vary in the T stage because of the sample shrinkage due to tissue dehydration and deformation during sectioning. In our study, 10 patients showed changes in the T stage. Specifically, seven cases initially classified as T1 were upgraded to T2, whereas three previously categorized as T2 were reclassified as T3 (Table 1). These findings indicate that micro-CT measurements offer superior predictive accuracy than pathological results. Hence, Lugol's iodine-enhanced micro-CT image can effectively reduce the insufficient measurement of DOI via simple pathological examination and provide more accurate measurements.

(A) 3D micro-computed tomography (CT) image (the red region was the tumor area). (B) Deepest radiological (r)-DOI measurement on micro-CT. (C) Pathological (p)-DOI measurement on pathological images. (D) r-DOI measurement on the micro-CT image corresponds to pathological images in (C).

**Table 1:** Patients’ characteristics and the change in T-stage

Patient N^o.^	Site of tumor	p-DOI	r-DOI	Difference	r-DOI (3D)	rT	pT
		(mm)	(mm)	(mm)	(mm)	(3D)	
1	Buccle	4.81	5.36	0.55	6.51	T2	T1
2	Tongue	4.15	4.28	0.13	4.46	T1	T1
3	Tongue	7.61	8.05	0.44	9.66	T2	T2
4	Buccle	4.26	4.35	0.09	5.34	T2	T1
5	Buccle	10.25	10.88	0.63	11.50	T3	T3
6	Tongue	3.91	4.32	0.41	4.87	T1	T1
7	Tongue	4.38	4.92	0.54	6.08	T2	T1
8	Tongue	4.76	5.47	0.71	6.78	T2	T1
9	Tongue	9.04	10.07	1.03	13.82	T3	T2
10	Tongue	10.17	10.89	0.72	14.07	T3	T3
11	Tongue	2.88	3.26	0.38	4.52	T1	T1
12	Tongue	13.50	14.22	0.72	15.46	T4a	T4a
13	Gingiva	8.60	9.53	0.93	10.03	T3	T2
14	Tongue	3.36	5.02	1.66	6.12	T2	T1
15	Gingiva	8.47	8.74	0.27	9.61	T3	T3
16	Buccle	4.88	5.03	0.15	5.1	T2	T1
17	Tongue	11.70	12.10	0.40	13.58	T4a	T4a
18	Tongue	6.04	6.19	0.15	6.92	T2	T2
19	Gingiva	5.31	5.71	0.40	7.6	T2	T2
20	Buccle	7.45	8.16	0.71	7.94	T2	T2
21	Tongue	6.68	7.67	0.99	8.43	T2	T2
22	Tongue	4.16	4.35	0.19	5.37	T2	T1
23	Tongue	9.49	10.30	0.81	10.82	T3	T2
24	Buccle	10.45	11.29	0.84	11.29	T3	T3
25	Tongue	6.75	7.71	0.96	8.55	T2	T2

### Correlation between r-DOI and p-DOI

**Figure 6:**
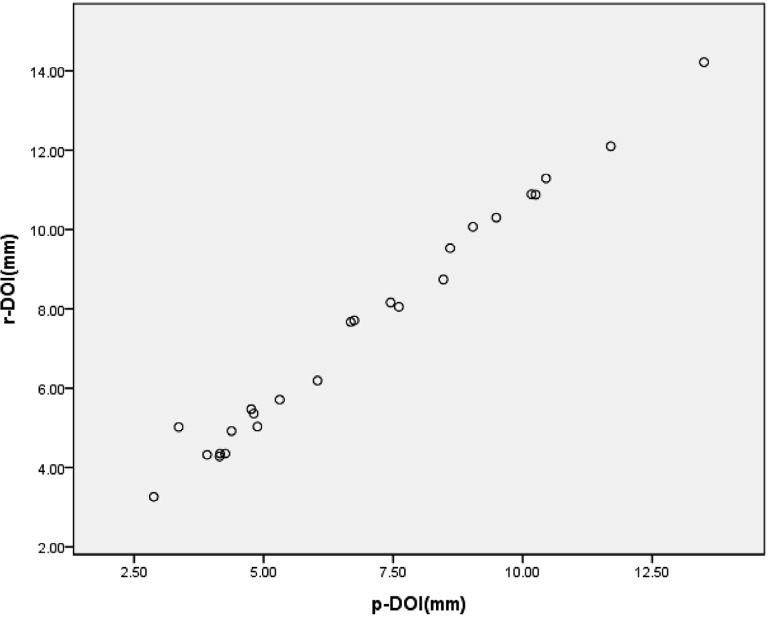
Correlation between p-depth of invasion (DOI) and r-DOI.

We observed a robust correlation between p-DOI and r-DOI (P<0.001), with a correlation coefficient of 0.986. The equation r-DOI = 1.03*p-DOI + 0.39 can express this relationship (Figure 6). This indicates that the tumor tissue changes slightly during tissue shrinkage. Therefore, surgeons can directly measure DOI using Lugol's iodine-enhanced micro-CT in the future.

## Discussion

According to the latest version of the NCCN guidelines, the DOI of OSCC offers the best predictor of occult lymph node metastasis and vascular lymphatic invasion.^15^ Recent prospective clinical research evidence shows that preventive neck dissection is strongly recommended for a DOI > 4 mm.^16^ DOI also configures a risk factor affecting oral cancer prognosis. Several recent clinical studies have shown that the deeper the infiltration of oral cancer, the worse the prognosis and the lower the survival rate.^17^ Tirelli, et al.^18^ (2018) retrospectively studied 167 patients with OSCC and analyzed the prognostic factors for survival and quality of life. They found that the five-year decision support system totaled 87.8% when the DOI was < 5 mm and patients’ decision support system equaled 68.2% when the DOI was > 5 mm, therefore the urgent need for effective inspection methods to accurately measure DOI. Our findings show the inherent advantages of micro-CT in three-dimensional inspection, effectively compensating for the limitations associated with conventional two-dimensional pathological sections when evaluating the DOI of OSCC. Numerous studies have compared and evaluated DOI measurements by different imaging examination methods, including CT,^19^ MRI,^20^ and ultrasound.^21^ CT examination^22^ poses problems, such as poor imaging contrast, susceptibility to interference from metal artifacts, and inability to capture the actual depth of tumor infiltration. MRI^23^ often overestimates the measured value of DOI^20^, and accurately evaluating lesions with DOI ≤ 5 mm is difficult.^24^ Although ultrasound can detect soft tissue lesions with a small DOI,^25^ its measurements have evident shortcomings. Noorlag, et al.^26^ (2020) performed ultrasound-assisted DOI measurements on 83 patients with T1-2 stage OSCC and reported that when the DOI was > 10 mm, ultrasound underestimated its true result. Moreover, the use of ultrasound probes to measure exophytic lesions is inevitable. Gentle pressure on the tumor can also cause discrepancies in measurements. This study chose micro-CT to accurately model, analyze, outline, and measure the region of interest with high resolution. Lugol'a iodine-enhanced micro-CT showed a fine structure inside the tissue and edge of the tumor. An effective tool for microstructural evaluation compensates for the shortcomings of these imaging modalities. Micro-CT can find the deepest DOI in 3D space, more accurately and reliably measuring than 2D pathological section data. Moreover, DOI by micro-CT can influence clinical T staging and holds great significance in guiding surgical decision-making. For example, a higher DOI is associated with an increased likelihood of lymph node metastasis, which, in turn, determines the necessity for elective or radical neck dissection.

The micro-CT in our experiment showed a resolution of 50 μm and a field of view of 50×40 mm. This resolution could discern the fundamental structure of the specimen. To achieve clearer imaging results, we employed resolutions of 25 and 10 μm, both of which showed superior imaging outcomes. This study maintained the field of view for the 25-μm resolution at 50×40 mm, decreasing that for the 10-μm resolution to 18×12 mm. Generally, as the field of view decreases, the resolution capability increases. Smaller fields of view may be more advantageous in achieving higher-resolution soft tissue imaging by micro-CT technology. Notably, limited resolution remains a common challenge when visualizing large-diameter soft-tissue specimens. Furthermore, due to the current limitations of CT software, this study restricted the assessment of micro-CT at a three-dimensional level to multiplanar reconstruction images. To enhance measurement accuracy, future studies should employ advanced CT software for analysis in three-dimensional tumor segmentation images.^27^

Although micro-CT can create high-resolution 3D models of *in vitro* tissues and provide anatomical information close to the histological resolution, it is unable to achieve cellular qualitative and quantitative analyses (including cell shape, nuclear and cytoplasmic features, and mitotic activity). However, the cell type and tumor cell-matrix relation pattern is crucial to determine the interaction between tumors and their microenvironment and their invasion potential. Moreover, owing to the irregular shape of salivary gland malignant tumors, blurred boundaries, cloud-like appearance, mostly multicenter lesions, and discontinuous masses, micro-CT images show grayscale values ranging from dense to non-dense areas. This makes it difficult to accurately distinguish the boundaries of salivary gland malignancies. Therefore, histopathological examinations are essential. The resolution of micro-CT is continuously improving and may reach the nanometer scale. This advancement is anticipated to surpass that of conventional pathological examination methods.

### Statements and declaration of ethical statement

Sample collection had the ethical approval by the Medical Ethics Committee of the Institute Affiliated Stomatology Hospital of Nanjing University Medical School (approval number: NJSH-2021NL-030). Informed consent was obtained from patients under standard ethical procedures.

### Informed consent

Written informed consent was obtained from patients for publication of this study and any accompanying images.

## Data Availability

The datasets generated during and/or analyzed during the current study are available in the SciELO Data repository, doi: https://doi.org/10.48331/scielodata.4NM6WS

## References

[1] Siegel RL, Giaquinto AN, Jemal A (2024). Cancer statistics, 2024. CA Cancer J Clin.

[2] Ebrahimi A, Gil Z, Amit M, Yen TC, Liao CT, International Consortium for Outcome Research (ICOR) in Head and Neck Cancer (2014). Primary tumor staging for oral cancer and a proposed modification incorporating depth of invasion: an international multicenter retrospective study. JAMA Otolaryngol Head Neck Surg.

[3] Tam S, Amit M, Zafereo M, Bell D, Weber RS (2019). Depth of invasion as a predictor of nodal disease and survival in patients with oral tongue squamous cell carcinoma. Head Neck.

[4] Lydiatt WM, Patel SG, O’Sullivan B, Brandwein MS, Ridge JA, Migliacci JC (2017). Head and Neck cancers: major changes in the American Joint Committee on Cancer eighth edition cancer staging manual. CA Cancer J Clin.

[5] Wang Y, Chen S, Ni Y, Magee D, Pu Y, Zhou Q (2018). Three-dimensional reconstruction with serial whole-mount sections of oral tongue squamous cell carcinoma: a preliminary study. J Oral Pathol Med.

[6] Mahajan A, Ahuja A, Sable N, Stambuk HE (2020). Imaging in oral cancers: a comprehensive review. Oral Oncol.

[7] Senter-Zapata M, Patel K, Bautista PA, Griffin M, Michaelson J, Yagi Y (2016). The role of micro-CT in 3D histology imaging. Pathobiology.

[8] Mccaul JA, Mcmahon JM, Quantrill J, Gilbert K, Mehanna HM, Shaw R (2017). LIHNCS: Lugol's iodine in head and neck cancer surgery: a multi-centre, randomised, controlled trial assessing the effectiveness of Lugol's iodine to assist excision of moderate dysplasia, severe dysplasia and carcinoma in-situ at mucosal resection margin of oral and oropharyngeal squamous cell carcinoma. J Clin Oncol.

[9] Xia CW, Gan RL, Pan JR, Hu SQ, Zhou QZ, Chen S (2020). Lugol's iodine-enhanced micro-CT: a potential 3-D imaging method for detecting tongue squamous cell carcinoma specimens in surgery. Front Oncol.

[10] Erazo-Puentes MC, Sánchez-Torres A, Aguirre-Urizar JM, Bara-Casaus J, Gay-Escoda C (2024). Has the 8th American joint committee on cancer TNM staging improved prognostic performance in oral cancer? A systematic review. Med Oral Patol Oral Cir Bucal.

[11] Lo Casto A, Cannella R, Taravella R, Cordova A, Matta D, Campisi G (2022). Diagnostic and prognostic value of magnetic resonance imaging in the detection of tumor depth of invasion and bone invasion in patients with oral cavity cancer. Radiol Med.

[12] Kaur J, Srivastava R, Borse V (2021). Recent advances in point-of-care diagnostics for oral cancer. Biosens Bioelectron.

[13] National Comprehensive Cancer Network (2018). Clinical Practice Guidelines in Oncology Head and Neck Cancers [Internet].

[14] Kukreja P, Parekh D, Roy P (2020). Practical challenges in measurement of depth of invasion in oral squamous cell carcinoma: pictographical documentation to improve consistency of reporting per the AJCC 8th Edition Recommendations. Head Neck Pathol.

[15] Tarsitano A, Del Corso G, Tardio ML, Marchetti C (2016). Tumor infiltration depth as predictor of nodal metastasis in early tongue squamous cell carcinoma. J Oral Maxillofac Surg.

[16] Melchers LJ, Schuuring E, Van Dijk BA, de Bock GH, Witjes MJ, van der Laan BF (2012). Tumour infiltration depth ≥4 mm is an indication for an elective neck dissection in pT1cN0 oral squamous cell carcinoma. Oral Oncol.

[17] Caldeira PC, Soto AM, Aguiar MC, Martins CC (2020). Tumor depth of invasion and prognosis of early-stage oral squamous cell carcinoma: a meta-analysis. Oral Dis.

[18] Tirelli G, Gatto A, Bonini P, Tofanelli M, Arnež ZM, Piccinato A (2018). Prognostic indicators of improved survival and quality of life in surgically treated oral cancer. Oral Surg Oral Med Oral Pathol Oral Radiol.

[19] Scotti FM, Stuepp RT, Dutra-Horstmann KL, Modolo F, Cavalcanti MG (2022). Accuracy of MRI, CT, and ultrasound imaging on thickness and depth of oral primary carcinomas invasion: a systematic review. Dentomaxillofac Radiol.

[20] Waech T, Pazahr S, Guarda V, Rupp NJ, Broglie MA, Morand GB (2021). Measurement variations of MRI and CT in the assessment of tumor depth of invasion in oral cancer: a retrospective study. Eur J Radiol.

[21] Caprioli S, Casaleggio A, Tagliafico AS, Conforti C, Borda F, Fiannacca M (2022). High-frequency intraoral ultrasound for preoperative assessment of depth of invasion for early tongue squamous cell carcinoma: radiological–pathological correlations. Int J Environ Res Public Health.

[22] Baba A, Ojiri H, Ogane S, Hashimoto K, Inoue T, Takagiwa M (2021). Usefulness of contrast-enhanced CT in the evaluation of depth of invasion in oral tongue squamous cell carcinoma: comparison with MRI. Oral Radiol.

[23] Murakami R, Shiraishi S, Yoshida R, Sakata J, Yamana K, Hirosue A (2019). Reliability of MRI-derived depth of invasion of oral tongue cancer. Acad Radiol.

[24] Li M, Yuan Z, Tang Z (2022). The accuracy of magnetic resonance imaging to measure the depth of invasion in oral tongue cancer: a systematic review and meta-analysis. Int J Oral Maxillofac Surg.

[25] Iida Y, Kamijo T, Kusafuka K, Omae K, Nishiya Y, Hamaguchi N (2018). Depth of invasion in superficial oral tongue carcinoma quantified using intraoral ultrasonography. Laryngoscope.

[26] Noorlag R, Klein Nulent TJW, Delwel VE, Pameijer FA, Willems SM, de Bree R (2020). Assessment of tumour depth in early tongue cancer: accuracy of MRI and intraoral ultrasound. Oral Oncol.

[27] Gomes JP, Costa AL, Chone CT, Altemani AM, Altemani JM, Lima CS (2022). Free three-dimensional image software in local extension assessment of oral squamous cell carcinoma: a pilot study. Braz J Otorhinolaryngol.

